# Profile of cervical cancer screening in Campo Grande, Mato Grosso do Sul, Brazil: an evaluative study, 2006-2018

**DOI:** 10.1590/S2237-96222022000200018

**Published:** 2022-08-29

**Authors:** Geize Rocha Macedo de Souza, Andrey Moreira Cardoso, Renata Palópoli Pícoli, Inês Echenique Mattos

**Affiliations:** 1 Secretaria Municipal de Saúde, Serviço de Doenças e Agravos não Transmissíveis, Campo Grande, MS, Brazil; 2 Fundação Oswaldo Cruz, Escola Nacional de Saúde Pública Sergio Arouca, Rio de Janeiro, RJ, Brazil; 3 Fundação Oswaldo Cruz, Campo Grande, MS, Brazil

**Keywords:** Uterine Cervical Neoplasms, Cervical Intraepithelial Neoplasia, Early Detection of Cancer, Women's Health, Time Series Studies

## Abstract

**Objective:**

To assess the coverage and quality of screening by the Cervical Cancer Control Program in Campo Grande, Mato Grosso do Sul, Brazil, between 2006 and 2018.

**Methods:**

This was a descriptive study of the cytology screening time series among women living in Campo Grande. A descriptive analysis of the demographic characteristics of these women and the quality of the tests performed in the last five years of the period was carried out. Temporal trends were analyzed using polynomial regression models.

**Results:**

578,417 cytology tests were recorded, of which 1.8% showed pre-malignant/malignant cytological changes. There was a 48.4% reduction in the number of tests performed in the Program's target age group. Test positivity varied between 2.2% and 3.3% and the percentage of unsatisfactory samples increased.

**Conclusion:**

The cervical cancer screening program has weaknesses that need to be overcome, such as low coverage of the target population, growth in the number of unsatisfactory samples and a low positivity rate.

**Study contributions**

**Main results**

Weaknesses were found in the Campo Grande Cervical Cancer Control Program (PCCCU): insufficient test provision, test periodicity not in line with needs, unsatisfactory quality of the samples collected and PCCCU information/system incompleteness.

**Implications for services**

There is a need for constant systematic evaluation of the health services provided for cervical cancer care, with the aim of meeting the PCCCU's goals and targets.

**Perspectives**

We hope that the findings of this study can contribute to more effectiveness of the screening of the population exposed to cervical cancer.

## Introduction

Cervical cancer is one of the most frequent tumors worldwide and an important cause of death among women.^1^ ^,^ ^2^ Globally, 528,000 new cases of cervical cancer and 266,000 deaths from the disease were estimated in 2012, which corresponds to annual age-adjusted incidence and mortality rates of 14/100,000 and 6.8/100,000 women, respectively.^1^ ^,^ ^2^ Due to the relevance of this public health problem, the World Health Organization has set global goals for addressing cervical cancer to be met between 2020 and 2030. One of the goals is to have achieved 90% treatment coverage during this period.^3^

Cervical cancer incidence and mortality distribution is heterogeneous globally.^4^ Whereas in developed countries progressive reduction in incidence and mortality can be seen, as a result of the effectiveness of population screening programs, in developing countries with greater social inequalities, these indictors remain at high levels, indicating the need to scale up access to screening, as well as early diagnosis and treatment.^4^ A total of 16,710 new cases of cervical cancer were registered in Brazil in 2020, so that this type of cancer is the third most frequent among females.^5^

Cervical cancer control actions fall under Women's Health, this being a strategic priority action area within the Brazilian National Health System (*Sistema Único de Saúde* - SUS), in particular at the Primary Health Care level.^6^ Diverse policies and programs involving cervical cancer control, such as the Comprehensive Women's Health Program (*Programa de Assistência Integral à Saúde da Mulher* - PAISM), have been developed since the 1980s, and have resulted in the establishment of 11 priorities within the Pact for Health (*Pacto pela Saúde*) (2006) with the objective of expanding coverage of preventive examinations and reducing cervical cancer mortality in Brazil.^6^

The main strategy for controlling cervical cancer consists of screening, which is based on the natural history of the disease and enables detection of precursor lesions, aiming at early treatment and non-progression of lesions to the invasive form. Coverage of the majority of the target population is an important factor in reducing cervical cancer incidence.^4^ ^,^ ^6^

A study that analyzed cervical cancer incidence and mortality in the state of Mato Grosso do Sul reported an increase of 139% in the incidence rate between 2001 and 2012, and growth of around 30% in age-adjusted mortality rates between 1979 and 2009.^7^ In that study, cervical cancer mortality rates were reported in the municipality of Campo Grande that ranged from 5.13 to 10.2/100,000 women between 1980 and 2009.^7^ In 2020, 270 new cases and an age-adjusted incidence rate of 18.3/100,000 women were estimated for the state of Mato Grosso do Sul as a whole, while 40 new cases and an age-adjusted incidence rate of 8.1/100,000 women were estimated for the state capital, Campo Grande.^8^ These data point to the importance of evaluating the actions of Campo Grande's Cervical Cancer Control Program (*Programa de Controle do Câncer do Colo do Útero* - CCPCU), to contribute to the development of screening actions contribute to the development of screening actions aimed at reducing cervical cancer incidence and mortality rates. 

The objective of this study was to evaluate the coverage and quality of cervical cancer screening in the municipality of Campo Grande, based on the records held on the Cervical Cancer Information System (*Sistema de Informação do Câncer do Colo do Útero* - SISCOLO) and the Cancer Information System (*Sistema de Informação do Câncer* - SISCAN).

## Methods

This descriptive ecological study analyzed the time series trend of cytology tests performed for cervical cancer screening in women residing in the municipality of Campo Grande, Mato Grosso do Sul, between 2006 and 2018. A descriptive analysis of the characteristics of the women assessed and the quality of the tests included on the cancer information system in the last five years of the period (2014-2018) was also performed.

Campo Grande, capital of the state of Mato Grosso do Sul, is located in the Midwest region of Brazil and has a territorial extension of 8,082 km^2^; its estimated general population is 916,001 inhabitants, while there are estimated to be 216,352 women in the 25-64 age group. The municipality's Primary Health Care network is formed of 58 Family Health Strategy centers, 11 Primary Health Care centers and three Family Clinics, which correspond to 74.6% network coverage in the capital. The databases used for the analysis of the time series defined by the study were the SISCOLO and the SISCAN, both responsible for registering cytology tests performed on the SUS.

Access to SISCOLO is public and for this study we accessed it online in January 2021. The analysis included all cervical cytology tests in women living in the city with data registered from 2006 to 2013, the last complete year available on this database. As of 2014, the cervical cytology test data began to be input to the SISCAN, a health information system to which, at the time this analysis was performed, there was no public access. The data for the period 2014-2018 were obtained from the SISCAN in January 2021, also online, following authorization by the Mato Grosso do Sul State Health Department.

The change from one information system to another during the study period resulted in differences in the configurations of variables related to the characteristics of women and to the quality of the tests on the respective databases. In order to carry out a time series analysis, a larger number of years is needed than the period available on the SISCAN (only five years). Thus, for the analysis of the cytology test series, we chose to use the available period (2006-2018), including the cytology test data held on the SISCOLO. However, in order to ensure the uniformity of the variables related to service users and the quality of tests, only one of the databases could be used, so in this case we only analyzed the data available on the SISCAN referring to a more recent period (2014-2018). 

In order to analyze the cytology test time series, we took the absolute number of cytology tests performed in each year and the absolute number of annual cytology tests performed on women in the target population (25 to 64 years old) by the PCCCU from 2006 to 2018, comprising a total of 578,417 cytology tests. The 25 to 64 age group is considered a priority because it presents a higher frequency of high-grade lesions, which, when treated early, do not progress to cervical cancer.^6^ Quantitative population data for the years covered by the study period were obtained from the SUS Information Technology Department (DATASUS).^9^ We used the variables available on the SISCAN (for the period 2014-2018) for residents in Campo Grande to perform descriptive analysis of the characteristics of the cytology tests and the population served.^10^

Initially, we evaluated the completeness of the variables available on the SISCAM, i.e. percentage completeness of these variables in the years covered by the study period, in order to select the variables to be analyzed. Based on the score proposed by Romero and Cunha (2006),^11^ the completeness of a variable was considered excellent when a percentage > 95% was found; good, when this percentage was between 90.1% and 95%; regular, between 80.1% and 90%; poor, between 50.1% and 80%; and very poor when it was ≤ 50%. The “age group”, “adequacy”, “representativeness of the transformation zone”, “within normality/cytological changes”, and “cytology” variables showed excellent and good completeness in all years of the study, and were therefore selected for analysis. The “prevention test period” variable showed poor completeness, evolving to regular at the end of the period; however, because it is an important variable for analysis, we chose to include it. The “schooling” variable was excluded from the analysis because its completeness was very low and was totally incomplete in the final years of the period. The “race/skin color” variable could not be evaluated because it was not among the variables available for filtering on the SISCAN system.

The following variables were therefore included in the study: age range (categorized in years: 24 or less; 25 to 64; ≥ 65); previous cytology test, not considering the time elapsed since the test (yes; no); prevention test period, consisting of the time elapsed between the performance of the previous cytology test and the performance of the current test (categorized into: same year; one year; two years; three years; four years or more); adequacy, i.e. classification of the quality of the sample collected (satisfactory; unsatisfactory; rejected); representativeness of the transformation zone (TZ), defined by the presence or absence of metaplastic and/or glandular epithelium in the sample (yes; no); cytological changes, i.e. tests with results that presented cellular atypia of interest for oncotic process or any benign change [categorized as: ASC-US (atypical squamous cells of undetermined significance, possibly non-neoplastic); ASC-H (atypical squamous cells of undetermined significance-cannot exclude high-grade epithelial lesion); non-neoplastic undetermined glandular atypia, non-neoplastic cells of unknown primary, and squamous cells with low-grade intraepithelial lesion; atypical glandular cells of undetermined significance-cannot exclude high-grade epithelial lesion; atypical cells of unknown primary-cannot exclude high-grade epithelial lesion]; squamous cells with high-grade intraepithelial lesion; squamous cells with high-grade intraepithelial lesion-cannot exclude microinvasion; and cancer (invasive epidermoid carcinoma, adenocarcinoma in situ, and invasive adenocarcinoma).^10^

The quality of the tests was evaluated by the adequacy of the sample, represented by the percentage of unsatisfactory samples. A sample is considered unsatisfactory when there is the presence of obscuring factors that hinder the evaluation of more than 75% of the epithelial cells,^12^ and when this happens, a new test is necessary. The indicator was calculated by the number of unsatisfactory samples divided by the total number of tests performed. TZ representativeness, characterized by the intersection of the lower cervix stratified epithelium and the upper cervix columnar columnar epithelium, was used as an indicator for the collection stage; the highest concentration of cytological change and cervical cancer precursor lesions are found in this area.^13^

The positivity rate informs as to the prevalence of cell changes in the tests, indicating the sensitivity of the screening process to detect lesions in the population examined. This indicator, in turn, was calculated by the sum of all cytology tests with changed results divided by the total number of satisfactory cytology tests and multiplied by 100. The Ministry of Health classifies positivity in cervical cancer screening as: very low, less than 2.0%; low, between 2.0% and 2.9%; expected, between 3% and 10%; and greater than expected, >10%.^14^ The “previous cytology” and “period” (in years) variables were used to assess whether the women had the test in accordance with recommended periodicity. 

Provision of tests was assessed by the indicator expressed by the “cytology tests / target population ratio” (female population aged 25 to 64 years) residing in Campo Grande, annually, between 2014 and 2018. Based on the recommendation to repeat the test every three years, the tests/target population ratio is expected to be 0.3 annually, in order to screen 100% of women in the priority age group.^15^ The following formula was used to calculate percentage change in the number of tests in the period from 2006 to 2018:

$$\frac{\left(No.\;\;of\;tests\;in\;2018\;-\;No.\;\;of\;tests\;in\;2006\right)}{No.\;\;of\;tests\;performed\;in\;2006\;x\;100}$$

For the purposes of the time series analysis, the absolute number of cytology tests performed in each year was taken to be the dependent variable (y), while the years of the study period were taken to be the independent variable (x). In the polynomial regression analysis, we estimated first-order (y = β0 + β1x), second-order (y = β0 + β1x + β2x^2^) and third-order (y = β0 + β1x + β2x^2^ + β3x^3^) polynomial models. The “year test performed” variable was centered in order to avoid data collinearity. The level of statistical significance (p-value = 0.05), the analysis of residuals, and the R2 value were taken as criteria for selecting the best model. This analysis was performed using SPSS20 software. The descriptive analysis of data on the characteristics of cytology tests and the population receiving care was carried out by means of absolute and relative distribution of variables, using Excel 2007. The analysis of the “previous cytology”, “period elapsed since previous test”, “adequacy”, “representativeness of the transformation zone” and “atypia” variables was carried out for three age groups: ≤ 24, 25 to 64 and ≥ 65 years.

The study project was approved by the Research Ethics Committee of the Escola Nacional de Saúde Pública Sergio Arouca/Fundação Instituto Oswaldo Cruz (CEP/ENSP/Fiocruz) as per Certificate of Submission for Ethical Appraisal No. 50454121.3.0000.5240. As the study used secondary data from the public domain, in which it is not possible to identify the participants, it was exempted from obtaining signed informed consent from the women whose cervical cancer screening cytology test data were analyzed.

## Results

During the study period, 578,417 cytology tests for women residing in Campo Grande were registered, 75.9% of which were performed on the 25 to 64 age group, this being the target population of the PCCCU. As of 2015, a substantial decrease in the number of registered tests was found. Highest PCCCU coverage was 19.5%, in 2008, subsequently falling to 7.5% in 2018. The lowest coverage in the period, 5.8%, occurred in 2017. Percentage change between the first and the last year of the time series was negative, both for the annual tests performed (-56.2%) and for the PCCCU target population (-48.4%) (Table 1).

**Table 1:** Total number of cytology tests, number of tests in the target population, and estimated percentage coverage of the Cervical Cancer Control Program in Campo Grande, Mato Grosso do Sul, Brazil, 2006-2018

Year	Number of tests		Number of tests in the target population (25-64 years)		Number of women in the target population and estimated percentage coverage (25-64 years)	
	n	%	n	%	n	%
2006	51,004	8.8	37,108	8.5	196,736	18.9
2007	50,146	8.7	36,675	8.4	202,421	18.1
2008	55,139	9.5	40,636	9.3	208,126	19.5
2009	55,047	9.5	41,026	9.3	213,771	19.1
2010	53,376	9.2	40,152	9.2	219,296	18.3
2011	56,255	9.7	42,798	9.8	224,087	19.1
2012	54,544	9.4	41,773	9.5	228,722	18.2
2013	44,129	7.6	34,103	7.8	233,209	14.6
2014	50,259	8.7	37,675	8.6	237,613	15.8
2015	34,073	5.9	25,172	5.7	241,969	10.4
2016	34,594	6.0	28,104	6.4	245,732	11.4
2017	17,501	3.0	14,458	3.3	249,383	5.8
2018	22,350	3.9	19,137	4.4	252,899	7.5
2006-2018	578,417	100.0	438,817	100.0	-	-
Change (%) (2006-2018)	-	-56.2	-	-48.4	-	-60.3

**Figure 1:**
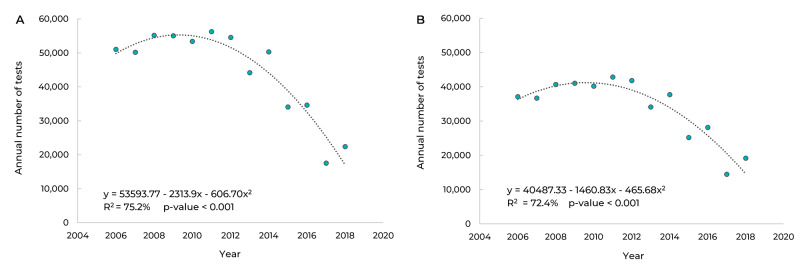
Trend of the annual number of cervical cytology tests in the general population (A) and in the target population (B) in Campo Grande, Mato Grosso do Sul, Brazil, 2006-2018

Figure 1 shows the evolution of the number of cytology tests performed in Campo Grande between 2006 and 2018. The linear model was the most appropriate for describing the series, as it explained 75.2% of the distribution with statistical significance (p-value < 0.001). The number of tests performed decreased, on average, by 2,313.90 in each year of the study period. Figure 1B shows the evolution of the number of cytology tests in Campo Grande, during the same period, considering only those performed in the PCCCU target population. Once again the linear model was the model that best represented the series, as it explained 72.4% of its distribution with statistical significance (p-value < 0.001). On average, the number of tests performed each year in this segment of the population decreased by 1,460.83.

In the period from 2014 to 2018, there was a predominance of tests performed in the 25 to 64 years age group of (78.4%), followed by women aged 24 years or less (15.7%) and those aged 65 years or more (5.8%). The cytology test ratio reached its highest value in 2014 (0.16); while falling in subsequent years, with ratios of 0.06 and 0.07 in 2017 and 2018, respectively, corresponding to a drop of more than 50% compared to 2014 (data not shown in tables or figures). 

In Table 2 it can be seen that most women who underwent cytology (92.4%) had already had at least one test in previous years; in the target population this accounted for 96.7%. As for cytology periodicity, we found a higher percentage for the last test being performed one year ago, in all age groups, corresponding to 54.9% in women under 25, 56.3% among women aged 25 to 64, and 54.3% in the group aged ≥ 65 years. 

**Table 2:** Percentage distribution of previous cytology tests and cytology tests per period, Cervical Cancer Control Program, Campo Grande, Mato Grosso do Sul, Brazil, 2014-2018

Previous cytology tests				Cytology tests per period					
Year	Yes	No	Total tests	Same year	1 year ago	2 years ago	3 years ago	4 years ago or more	Total tests
	n (%)		n	n (%)					n
**≤ 24 anos**									
2014	5,906 (67.3)	2,867 (32.7)	8,773	758 (13.2)	3,076 (53.6)	1,247 (21.7)	417 (7.3)	237 (4.2)	5,735
2015	4,465 (70.2)	1,894 (29.8)	6,359	407 (9.4)	2,748 (63.2)	830 (19.1)	266 (6.1)	95 (2.2)	4,346
2016	3,071 (72.0)	1,197 (28.0)	4,268	238 (8.0)	1,616 (54.2)	841 (28.2)	210 (7.0)	78 (2.6)	2,983
2017	1,369 (72.5)	520 (27.5)	1,889	83 (6.3)	654 (49.2)	403 (30.3)	158 (11.9)	31 (2.3)	1,329
2018	1,421 (71.7)	560 (28.3)	1,981	92 (6.7)	554 (40.5)	445 (32.5)	183 (13.4)	95 (6.9)	1,369
**2014-2018**	16,232 (69.8)	7,038 (30.2)	23,270	1,578 (10.0)	8,648 (54.9)	3,766 (23.9)	1,234 (7.8)	536 (3.4)	15,762
**25-64 years**									
2014	33,022 (95.9)	1,421 (4.1)	34,443	3,055 (9.7)	17,766 (56.1)	7,425 (23.5)	2,261 (7.1)	1,140 (3.6)	31,647
2015	23,039 (97.1)	690 (2.9)	23,729	1,534 (7.0)	14,334 (64.8)	4,405 (19.9)	1,331 (6.0)	512 (2.3)	22,116
2016	25,653 (96.8)	844 (3.2)	26,497	1,497 (6.1)	13,896 (56.3)	7,270 (29.4)	1,498 (6.1)	525 (2.1)	24,686
2017	13,344 (97.3)	375 (2.7)	13,719	639 (4.9)	6,999 (54.4)	3,618 (28.1)	1,346 (10.5)	270 (2.1)	12,872
2018	17,694 (97.2)	513 (2.8)	18,207	762 (4.5)	8,028 (47.0)	5,460 (31.9)	1,951 (11.4)	890 (5.2)	17,091
**2014-2018**	112,752 (96.7)	3,843 (3.3)	116,595	7,487 (6.9)	61,023 (56.3)	28,178 (26.0)	8,387 (7.7)	3,337 (3.1)	108,412
**≥ 65 years**									
2014	2,391 (92.4)	196 (7.6)	2,587	195 (8.6)	1,195 (53.0)	560 (24.9)	189 (8.4)	114 (5.1)	2,253
2015	1,883 (95.9)	81 (4.1)	1,964	125 (7.0)	1,117 (62.7)	371 (20.8)	128 (7.2)	41 (2.3)	1,782
2016	1,689 (96.3)	65 (3.7)	1,754	105 (6.6)	876 (55.1)	471 (29.7)	100 (6.3)	36 (2.3)	1,588
2017	912 (96.7)	31 (3.3)	943	42 (4.9)	465 (53.9)	252 (29.2)	86 (10.0)	17 (2.0)	862
2018	988 (96.7)	34 (3.3)	1,022	59 (6.2)	387 (40.7)	311 (32.7)	126 (13.2)	68 (7.2)	951
**2014-2018**	7,863 (95.1)	407 (4.9)	8,270	526 (7.1)	4,040 (54.3)	1,965 (26.4)	629 (8.5)	276 (3.7)	7,436

The proportion of unsatisfactory samples in the period from 2014 to 2018 ranged between 0.7% and 1.8% in the target population, 0.8% and 1.6% in women under 25 years old, and between 1.3% and 2.6% in those aged ≥ 65 years. A total of 29 samples were rejected, 20 of which related to women aged 25 to 64 years. As for the representativeness of the TZ, we found that in the target population the TZ was absent in 51.0% of the samples collected. In women under 25 years of age, the percentage of samples without the TZ ranged between 38.9% and 49.5%, and in those aged 65 years or more, it ranged between 67.7% and 73.7% (Table 3). 

**Table 3:** Percentage distribution of cervical cytology tests according to adequacy and transformation zone, Cervical Cancer Control Program, Campo Grande, Mato Grosso do Sul, Brazil, 2014-2018

Age group (in years)	Year	Adequacy				Transformation zone		
		Satisfactory	Unsatisfactory	Rejected	Total tests	Yes	No	Total tests
		n (%)			n	n (%)		n
**≤ 24**	2014	9,540 (99.2)	78 (0.8)	1 (0.0)	9,619	5,316 (55.7)	4,223 (44.3)	9,539
	2015	6,665 (99.0)	64 (1.0)	1 (0.0)	6,730	3,365 (50.5)	3,303 (49.5)	6,668
	2016	4,457 (98.7)	56 (1.3)	2 (0.0)	4,515	2,710 (60.7)	1,755 (39.3)	4,465
	2017	1,990 (98.4)	32 (1.6)	- (0.0)	2,022	1,219 (61.1)	777 (38.9)	1,996
	2018	2,085 (98.6)	29 (1.4)	- (0.0)	2,114	1,148 (54.7)	952 (45.3)	2,100
**2014-2018**		24,737 (98.9)	259 (1.1)	4 (0.0)	25,000	13,758 (55.5)	11,010 (44.5)	24,768
**25 a 64**	2014	37,386 (99.2)	284 (0.8)	5 (0.0)	37,675	18,402 (49.2)	18,983 (50.8)	37,385
	2015	24,977 (99.2)	190 (0.8)	5 (0.0)	25,172	11,330 (45.3)	13,662 (54.7)	24,992
	2016	27,861 (99.1)	239 (0.9)	4 (0.0)	28,104	14,691 (52.6)	13,217 (47.4)	27,908
	2017	14,200 (98.2)	254 (1.8)	4 (0.0)	14,458	7,367 (51.7)	6,883 (48.3)	14,250
	2018	18,897 (98.8)	238 (1.2)	2 (0.0)	19,137	8,759 (46.2)	10,189 (53.8)	18,948
**2014-2018**		123,321 (99.0)	1.205 (1.0)	20 (0.0)	124,546	60,549 (49.0)	62,934 (51.0)	123,483
**≥ 65**	2014	2,917 (98.4)	47 (1.6)	1 (0.0)	2,965	825 (28.3)	2,091 (71.7)	2,916
	2015	2,139 (98.6)	29 (1.3)	3 (0.1)	2,171	568 (26.5)	1,574 (73.5)	2,142
	2016	1,941 (98.3)	33 (1.7)	1 (0.0)	1,975	609 (31.3)	1,339 (68.7)	1,948
	2017	994 (97.4)	27 (2.6)	- (0.0)	1,021	325 (32.3)	680 (67.7)	1,005
	2018	1,079 (98.2)	20 (1.8)	- (0.0)	1,099	284 (26.3)	797 (73.7)	1,081
**2014-2018**		9,070 (98.3)	156 (1.7)	5 (0.0)	9,231	2,611 (28.7)	6,481 (71.3)	9,092

Percentage cellular atypia was higher in those aged 24 years or less (4.4%); it was 2.3% in the samples performed on the PCCCU target age group. The highest percentage of high-grade lesions was found in the target population, although it accounted for less than 1.0%. The highest number of cancer cases (23) was also found in the group of women aged 25 to 64 years old. Annual change in the positivity rate ranged between 2.2% and 3.3% in the target population (Table 4).

**Table 4:** Percentage distribution of cervical cytology result changes by age group, Cervical Cancer Control Program, Campo Grande, Mato Grosso do Sul, Brazil, 2014-2018

Year	Atypical	High-grade squamous cell intraepithelial lesion	High-grade squamous cell intraepithelial lesion-cannot exclude microinvasion	Cancer	Changed test results	Satisfactory test results	Positivity rate	tests
	%							n
**≤ 24 years**								
2014	450 (4.7)	34 (0.3)	- (0.0)	1 (0.0)	485 (5.0)	9,540 (99.2)	5.1	9,619
2015	240 (3.6)	17 (0.2)	- (0.0)	1 (0.0)	258 (3.8)	6,665 (99.0)	3.9	6,730
2016	198 (4.4)	13 (0.3)	- (0.0)	- (0.0)	211 (4.6)	4,457 (98.7)	4.7	4,515
2017	102 (5.0)	6 (0.3)	1 (0.0)	- (0.0)	109 (5.4)	1,990 (98.4)	5.5	2,022
2018	118 (5.6)	12 (0.6)	- (0.0)	- (0.0)	130 (6.1)	2,085 (98.6)	6.2	2,114
**2014-2018**	1,108 (4.4)	82 (0.3)	1 (0.0)	2 (0.0)	1,193 (4.8)	24,737 (98.4)	0.0	25,000
**25-64 years**								
2014	866 (2.3)	123 (0.3)	10 (0.0)	8 (0.0)	1,007 (2.7)	37,386 (99.2)	2.7	37,675
2015	443 (1.8)	79 (0.3)	12 (0.0)	5 (0.0)	539 (2.2)	24,977 (99.2)	2.2	25,172
2016	672 (2.4)	120 (0.4)	12 (0.0)	7 (0.0)	811 (2.9)	27,861 (99.1)	2.9	28,104
2017	410 (2.8)	55 (0.4)	8 (0.0)	2 (0.0)	475 (3.3)	14,200 (98.2)	3.3	14,458
2018	513 (2.7)	89 (0.5)	13 (0.1)	1 (0.0)	616 (3.2)	18,897 (98.7)	3.2	19,137
**2014-2018**	2,904 (2.3)	466 (0.4)	55 (0.0)	23 (0.0)	3,448 (2.8)	123,321 (99.0)	0.0	124,546
**≥ 65 years**								
2014	40 (1.3)	12 (0.4)	- (0.0)	2 (0.1)	54 (1.8)	2,917 (98.4)	1.8	2,965
2015	43 (2.0)	4 (0.2)	2 (0.1)	1 (0.0)	50 (2.3)	2,139 (98.5)	2.3	2,171
2016	32 (1.6)	7 (0.3)	- (0.0)	4 (0.2)	43 (2.2)	1,941 (98.3)	2.2	1,975
2017	26 (2.5)	4 (0.4)	1 (0.1)	4 (0.4)	35 (3.4)	994 (97.3)	3.5	1,021
2018	23 (2.1)	2 (0.2)	1 (0.1)	- (0.0)	26 (2.4)	1,079 (98.2)	2.4	1,099
**2014-2018**	164 (1.8)	29 (0.3)	4 (0.0)	11 (0.1)	208 (2.2)	9,070 (98.3)	0.0	9,231

## Discussion

This study found a reduction in the number of cytology tests performed for cervical cancer screening, including screening in the PCCCU target population, in the period 2006-2018. Moreover, a low cytology/population ratio was identified in all the years of the study, as well as a high percentage of previous cytology and tests repeated in a period of less than one year. There was a predominance of satisfactory samples in all age groups; however, TZ representativeness below 50% was found in the priority age group, namely 25 to 64 years old, as well as a high percentage of atypia. A low positivity rate (2.0% to 2.9%) was found in the target population in the period 2014-2016, while it was within the expected range (3.0% to 10%) in 2017 and 2018.

In Brazil, a goal of 85% has been set for cytology test coverage in the target population by 2022.^16^ Based on the findings of this study, Campo Grande has not reached this level yet. This low cytology coverage shows a weakness in the cervical cancer screening program in the municipal health network and could be related in part to occasional unavailability of supplies for the test, as documented in the municipality in that period.^17^ However, it must be emphasized that part of the women living in Campo Grande possibly had cytology tests in the city's supplementary health network and, if these data were recorded on the information system, population coverage would certainly reach a higher value. Oliveira et al.,^18^ when comparing self-reported data from the National Health Survey (*Pesquisa Nacional de Saúde* - PNS) and the Chronic Disease Risk and Protective Factors Surveillance Telephone Survey (*Sistema de Vigilância de Fatores de Risco e Proteção para Doenças Crônicas por Inquérito Telefônico* - VIGITEL), both referring to 2013, found that coverage of cytology tests for cervical cancer prevention in Campo Grande was around 87% among women in the PCCCU target age group who reported having had at least one test in the last three years.^16^

A study conducted in 17 European Union countries, with data from 2004 to 2014, showed great variability in percentage coverage of cervical cancer preventive screening, with no program reaching the goal of 85% defined by the European guidelines.^19^ Sweden, the United Kingdom and Norway had the highest coverage, close to 80%, while Slovakia and Italy had the lowest coverage, with values around 20% and 40%, respectively.^19^

The cytology/population ratio we found did not reach the value set (0.62) by Campo Grande for the year 2018,^20^ indicating a shortfall in the provision of tests in the municipal network. However, this indicator should also be analyzed with caution, since this study used data from SISCAN related only to tests performed in the public health network and therefore it could be underestimated. Be that as it may, the finding indicates that the primary health care network is apparently not reaching part of its clientele. Lopes and Ribeiro,^21^ in a literature review in which limiting and facilitating factors for the control of cervical cancer were analyzed, point out, among the limiting factors of access related to health service management and/or health professionals, low provision of services, shortage of human resources, overcrowding, and poor welcoming at and linkage with health centers, among other factors. 

Furthermore, not having cytology tests may be associated with individual issues, such as fear of the test itself or of the possible diagnosis, anxiety, shame, low schooling,^16^ ^,^ ^18^ ^,^ ^19^ as well as with some race/skin color categories.^16^ ^,^ ^18^ ^,^ ^19^ ^,^ ^21^ ^,^ ^22^ According to this study, 3.3% of women from Campo Grande reported not having had previous cytology before their current test. This value is below that found in a study conducted in the municipality of Chapecó, Santa Catarina (11.6%), with data from SISCAN referring to 2015.^13^ As for screening periodicity, there was a high concentration of tests with an interval of up to one year between one sample collection and another in the target population, which suggests unnecessary repetitions, to the detriment of reaching other women who could benefit from access to cervical cancer screening. These findings differ from the recommendations of the Brazilian Guidelines for Cervical Cancer Screening (*Diretrizes Brasileiras para o Rastreamento de Câncer de Colo do Útero*) (2016), which recommend performing two cytology tests in the first year of testing and, if both results are negative, a new test every three years.^6^ Measures that ensure access to women who have never had the test, with timely collection, need to be emphasized. 

In order to achieve adequate diagnosis of a cytology test, the smear must be satisfactory, i.e. there must be a large number of squamous and glandular cells. They must be well distributed and fixed, and must contain the TZ, the region in which more than 90% of cervical cancer precursor lesions are found.^13^ In the present study, absence of the ZT was found in more than 50% of the cytology tests performed in the target population, increasing to more than 67% in the group aged 65 years or older, which may suggest technical shortcomings among the professionals responsible for sample collection. In the Chapecó study,^13^ the authors reported TZ absence in 24.3% of tests, which was lower than the percentage found in Campo Grande. The absence of these two epithelia may contribute to false-negative results and cause delay in the diagnosis of cervical cancer precursor lesions. Adequate collection of cytology test samples, when correlated with 80% population coverage, could reduce cervical cancer incidence by up to 90%.^13^

The highest frequency of low-grade lesions occurs before 25 years of age, and most of them regress spontaneously.^6^ After 64 years of age, in the case of women who have had regular screening, the likelihood of developing cervical cancer is reduced, since its evolution is slow.^6^ The findings of the present study were compatible with these statements, since we found a higher percentage of high-grade lesions in women aged 25 to 64 years, this being the age range recommended for screening this form of cancer in Brazil.^6^

The main cytological changes found in this study, for all age groups, were ASCs, with a higher concentration in young women (24 years old or younger). This indicator is related to laboratory quality and it is expected that only 3% to 5% of all cytology tests be classified as ASCs.^23^ In the target population, this indicator corresponded to 2.3% in the study period, showing a small increase in the last two years. A high percentage of ASCs may conceal results of greater concern, considering that 20% to 40% of women who present ASCs may develop low-grade lesions, and 5% to 15% may develop high-grade lesions.^23^

Regarding cell changes, the target population showed a higher percentage of high-grade intraepithelial lesions. These changes require follow-up, and the current recommendation is to perform specific tests for screened women, such as colposcopy, new cytology, biopsy, and/or type 1, 2, or 3 excisions - depending on how the service is organized.^24^

The positivity rate indicates the prevalence of cell changes in cytology tests, as well as the sensitivity of the service provider in detecting lesions in the population examined. The positivity rate is classified by the Ministry of Health as follows: very low (less than 2%); low (between 2% and 2.9%); expected (between 3% and 10%); and greater than expected (more than 10%).^14^ ^,^ ^25^ This study found a variation in the positivity rate, with predominance of the “low” and “expected” categories in the target population. This is a warning to service providers regarding follow-up. In the United Kingdom, which has a well-structured screening program, the positivity rate was 6.4% in 2015,^26^ almost double that found in Campo Grande in this study. Dias et al.,^27^ when analyzing data from the SISCOLO for the period 2002-2006, found an increase of 22.9% in Brazil as a whole, although with variations between the country's macro-regions. A study conducted in Piauí, with data from 2006-2013, found a low positivity rate (2.2%) in women aged 65 years or older, and a very low rate (1.5%) in women under 25 years old.^28^ Another study conducted in Minas Gerais, with data from 2006-2011, found a very low rate (less than 2.0%),^29^ which could indicate false-negative test results. In order to improve this rate, it would be important to ensure continuing education for health professionals involved in the different stages of the process, with the aim of ensuring quality in the interpretation of cytology results.^30^

This study has limitations. The tests included were only those performed within the public health system, corresponding, therefore, to a partial view of the reality of the PCCCU in Campo Grande. It is also possible that weaknesses found in the PCCCU information systems used as a source of data, i.e. the information reported here, have caused biases in the characterization of the local situation.

The “schooling” variable, associated in the literature with women not testing,^16^ ^,^ ^18^ ^,^ ^19^ could not be explored in this study due to the incompleteness of this information in the PCCCU. Likewise, it was not possible to evaluate categories of race/skin color that are socially more vulnerable and have lower percentages of adherence to cervical cancer screening,^16^ ^,^ ^21^ ^,^ ^22^ due to the unavailability of this variable for analysis on the SISCAN. It is noteworthy that this variable is relevant in the municipality we studied, given the high percentage of indigenous people in Campo Grande, who live in conditions of socioeconomic disadvantage and marginalization.^20^

However, it was possible to identify important weaknesses in the PCCCU in Campo Grande, especially the insufficient provision of testing, the periodicity of testing, which is lower than necessary, and the unsatisfactory quality of the samples collected. Moreover, the use of the PCCCU information systems as sources of data in this study enabled identification of problems regarding incompleteness of the variables collected, demonstrating the need for improvement of this tool, which is indispensable to management and an important source of data for epidemiological studies.
